# Parasitic fauna of Eurasian beavers (*Castor fiber*) in Sweden (1997–1998)

**DOI:** 10.1186/s13028-021-00588-w

**Published:** 2021-06-02

**Authors:** Per-Arne Åhlen, Göran Sjöberg, Margareta Stéen

**Affiliations:** 1 Department of Wildlife, Fish and Environmental Studies, Faculty of Forest Sciences, Swedish University of Agricultural Sciences, 90183 Umeå, Sweden; 2 Department of Anatomy, Physiology and Biochemistry, Faculty of Veterinary Medicine and Animal Science, Swedish University of Agricultural Sciences, PO. Box 7011, 750 07 Uppsala, Sweden; 3 Present Address: Swedish Association for Hunting and Wildlife Management, Öster Malma, 611 91 Nyköping, Sweden

## Abstract

**Background:**

The parasitic fauna of beavers (*Castor fiber* and *C. canadensis*) has been well studied in many parts of their respective areas of distribution. In Scandinavia there have, however, been limited investigations conducted on the parasites of beavers in recent times. The present study is the first quantitative survey of parasites on beavers living in Sweden and elsewhere in Scandinavia. We investigated the parasitic fauna of the Eurasian beaver (*C. fiber*) in a North–South gradient in Sweden. The aim of the study was to investigate parasite distribution and prevalence in particular, related to average yearly air temperature and different age groups of beavers. A total of 30 beavers were sampled at eight localities, spanning a 720 km North–South gradient during the springs of 1997 and 1998.

**Results:**

Five parasite taxa were identified. Four of these were present in all of the examined beavers, *Stichorchis subtriquetrus* (trematode), *Travassosius rufus* (nematode), *Platypsyllus castoris* (coleopteran), and *Schizocarpus* spp*.* (arachnid). A higher number of new infections of *S. subtriquetrus,* and more adults of *T. rufus,* were seen in beavers in southern Sweden where temperatures are higher. One-year old beavers had a higher infestation of *S. subtriquetrus,* but not of *T. rufus*, than older individuals.

**Conclusions:**

The parasite fauna of Swedish beavers mirrored the impoverished parasite fauna of the original Norwegian population, and the high prevalence of parasites could be due to low major histocompatibility complex (MHC) polymorphism. Young beavers had a higher load of trematodes, probably depending on behavioural and ecological factors. Warmer temperatures in southern localities likely contributed to increased endoparasite loads.

## Background

The Eurasian beaver (*Castor fiber*) is native to Europe and northern Asia, and the North American beaver (*Castor canadensis*) to North America. *Castor canadensis* has been introduced to other localities, including Europe. Eurasian beavers were severely reduced in numbers over the course of many centuries and, by the early twentieth century, only about 1200 individuals remained in scattered refugia across Europe and Asia, including southern Norway [1]. In Sweden, as in many other countries, the beaver had become extinct. Following protection in areas where it remained, the Eurasian beaver was reintroduced and re-established in many parts of its former range of distribution [1]. The species was first re-established in regions of the Soviet Union, then in other parts of northern and eastern Europe and later in western and southern Europe. More recently there have been projects for reintroduction to Scotland and other parts of Great Britain, such as the Scottish Beaver Trial program [1, 2].

**Fig. 1:**
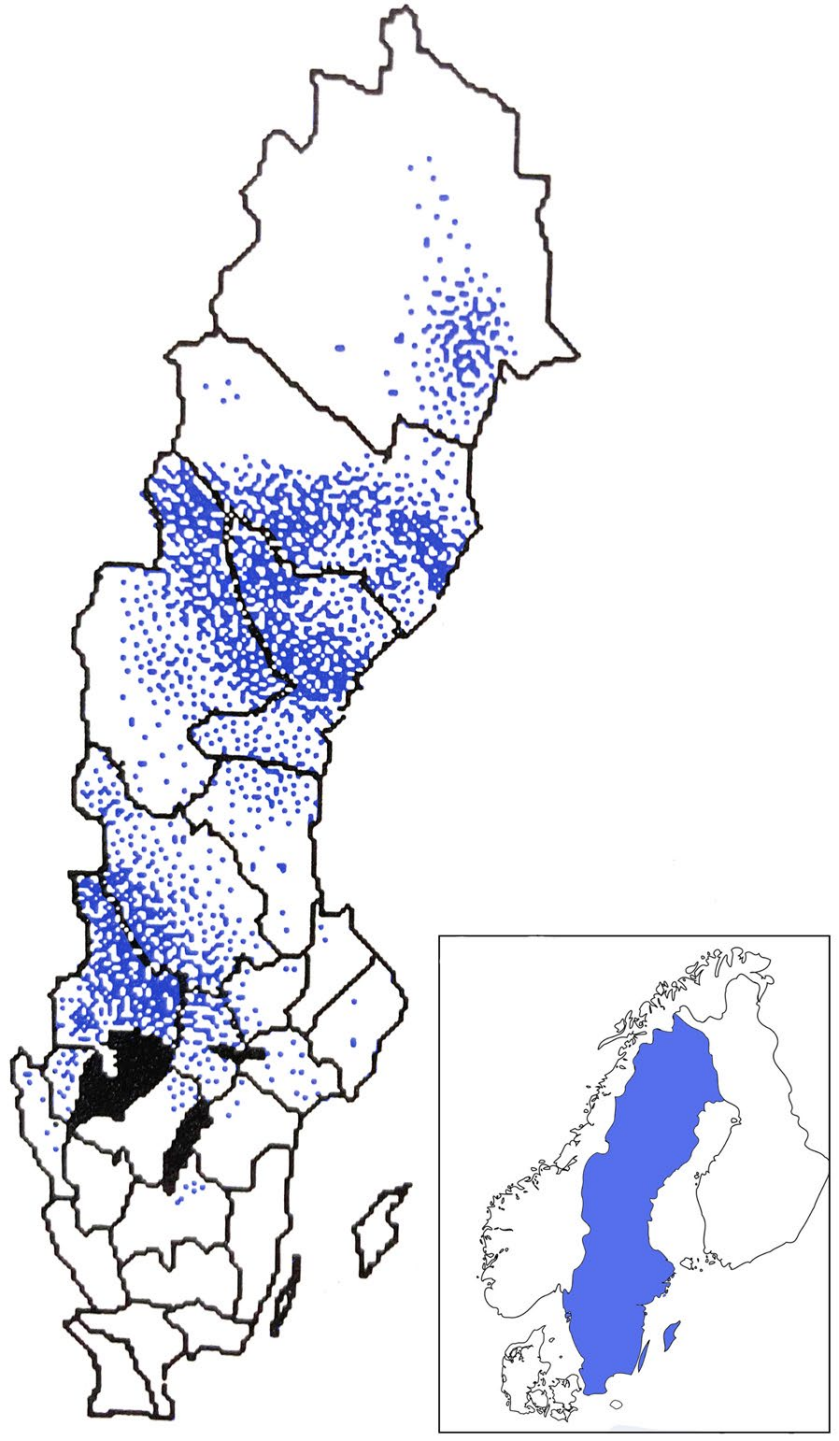
The distribution of beavers in Sweden 1992. Map adapted after Göran Hartman [3]. Inset of the Nordic countries shows the position of Sweden

The Norwegian *C. fiber* population is the origin of the Swedish population. Reintroduction to Sweden started in the 1920s following extinction through hunting in the 1870s [3]. A small number of beavers, about 80 individuals, were trapped from the remnant population in South-East Norway to re-establish the population in Sweden, however, likely, no more than 46 individuals were successfully introduced [3, 4]. The Norwegian population had been subjected to a bottleneck constraint. The beavers translocated to Sweden then underwent the same constraints when released in small separated groups. After a lag phase during the first decades, the Swedish beaver population increased rapidly during the 1970’s, and by the 1990’s it was estimated at more than 100,000 individuals and had a distribution covering a large part of the country [3] (Fig. 1). The populations of Eurasian beavers have generally increased rapidly since the late nineteenth century. However, genetic studies show that the genetic diversity within current populations vary substantially. In the Fennoscandian populations, with Norwegian origin, the genetic diversity is considerably lower as compared with other populations [4, 5].

Introducing new species can lead to transmission of pathogens or parasites to native species, causing a great economic and ecological threat to valuable native species [6]. It is therefore essential to know what parasites and/or diseases are transmitted by introducing new species to new localities. This may also be true for reintroductions for conservation purposes. Already in the 1960s there were warnings that necessary measures for preventing the transfer of parasites with their specific host (beaver in this case) from native localities into new environments should be considered [7]. One case, involving beavers from Bavaria, Germany, is the import of the tapeworm *Echinococcus multilocularis*—not a typical parasite of beavers—into Great Britain, where this parasite does not officially occur. One of these beavers, which had been wild-caught, and transported to the UK where it had died as captive, was screened by British laboratories, and lesions in the liver were found to be positive for *E. multilocularis* using both histological and polymerase chain reaction (PCR) methods [8]. In the International Union for Conservation of Nature (IUCN) guidelines for reintroductions and other conservation translocations, it is stressed that a risk assessment for parasites and diseases needs to be made when planning reintroductions [9]. Conservation biologists must also take into account the biodiversity of parasites [10, 11].

**Fig. 2:**
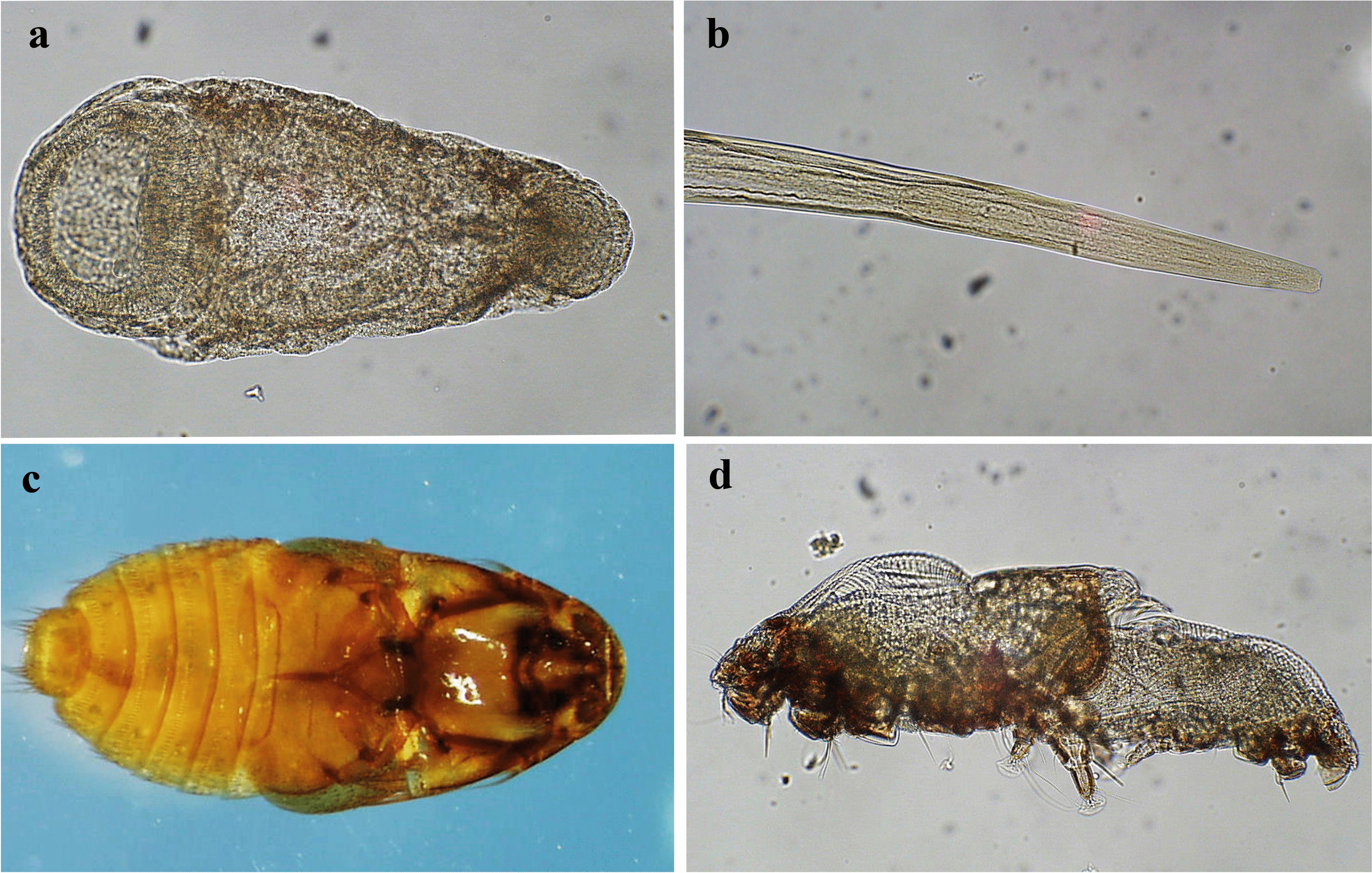
The parasites found in the studied beavers. **a** Metacercarian of the beaver fluke *Stichorchis subtriquetrus*; **b** Anterior part of the nematode *Travassosius rufus*; **c** Ventral view of the beaver beetle *Platypsyllus castoris*; **d** beaver mite *Schizocarpus* spp. All photos by Johan Höglund, Department of Biomedical Science and Veterinary Public Health; Parasitology Unit, Swedish University of Agricultural Sciences, Uppsala, Sweden

The parasitic fauna of beavers is well investigated in most of their distribution areas. In North America, several surveys cover both the helminth and arthropod parasites of *C. canadensis* [12–18]. In Europe also, a number of studies of the parasites of *C. fiber* are reported [7, 19–35]. In total, 33 helminth species have been found in *C. fiber* [7, 36, 37]. The trematode *Stichorchis subtriquetrus* (Fig. 2a) is the dominant parasite in the Eurasian beaver as well as in *C. canadensis* in North America. *Stichorchis subtriquetrus* has an indirect lifecycle with snails as intermediate hosts [18, 37]. Among obligate nematodes, *T. rufus* (Fig. 2b) is common in *C. fiber* in Europe, while *Travassosius americanus* and *Castorstrongylus castoris* are the predominant species in *C. canadensis* in North America. Another nematode, *Tricocephalus castoris*, is found in beavers only in the river Elbe in Germany [7]. These nematodes have a direct life cycle [38]. *Travassosius rufus* and *S. subtriquetrus* are species described as adapted specifically for the genus *Castor* [36].

The first discovery of the beaver beetle, *Platypsyllus castoris* (Fig. 2c), was in 1869, and the first certain manifestation of the species on *C. fiber* was in the Camargue, France, in the year 1884 [39]. In Sweden *P. castoris* was first detected in 1912 on a dead beaver found floating in the sea off the West coast [40]. The beaver was at this time regarded as extinct in Sweden and the origin of the individual is unknown. The first specimens of *P. castoris* from a Swedish beaver were collected in 1938 by Wirén, from a beaver killed by dogs [41]. Detailed accounts of *P. castoris* prevalence are scarce but, in a North American study, adults were found in over 60% of 45 living and dead beavers [42]. The distribution of *P. castoris* is, as far as known, over its total range identical with the beaver species [43]. The species has earlier been regarded as an ectoparasite [25, 43] but was recently labelled an obligate commensal with the two beaver species, with no adverse effects for the hosts [26]. To date, 39 species of beaver mites, *Schizocarpus* spp., are known from the Eurasian beaver and more than ten mite species, inhabiting different fur zones, can simultaneously parasitize an individual host (Fig. 2d). Several species inhabit the fur of the head [44–47].

Norwegian beavers were examined during the 1920s, in the London Zoo and in Latvia, and the only parasites found were *S. subtriquetrus* and *T. rufus* [7]. Parasites were also checked for in a beaver trapped in Norway in 1976, and the same two species of parasites were found [48]. As part of the Scottish Beaver Trial a health surveillance program was established in 2008, based on IUCN guidelines [2, 9]. Sixteen beavers, trapped in Norway, were screened at least once before they were released in Knapdale, Scotland. In three out of six beavers that died during quarantine, and in an additional five out of 16 beavers, *S. subtriquetrus* was detected along with *T. rufus*. The death of the beavers was, however, not linked to these parasites. *Stichorchis subtriquetrus* is a parasite specific to beavers and not pathogenic under normal circumstances so, in accordance with the IUCN guidelines [9], the beavers were not dewormed before release [2, 49]. The same conclusion was apparently reached for *T. rufus*. *Stichorchis subtriquetrus* had already been recorded from free-ranging beavers [32], since there have been unofficial releases of beavers of mixed origins elsewhere in Scotland [50]. *Platypsyllus castoris* has accidentally been reintroduced via the Scottish Beaver Trial [26] and the species has also elsewhere been recognized as a co-reintroduction with beavers [51].

It is known [52, 53] that animal populations tend to lose parasites when a bottleneck manifests. Parasite hosts introduced to new habitats also tend to harbour a subset of their original parasite fauna [54]. It could therefore be expected that beavers in Sweden should show the same impoverished parasite fauna as in the relict Norwegian beaver population, reflecting the aforementioned bottleneck situation and reintroduction of the species into Sweden [3].

It has been suggested that mammal species, or populations with low major histocompatibility complex (MHC) polymorphism, may be particularly vulnerable to infection [55–58]. The MHC plays a crucial role in the vertebrate immune system. The high levels of polymorphism normally found in natural populations are important to ensure long-term survival [59]. It has been shown that for most relict *C. fiber* populations, important MHC genes are specific and monomorphic [60], possibly increasing the risk for epizootics. Because of the bottlenecks that the Swedish beaver population has undergone, and the documented low genetic diversity in Swedish beavers [4], a high parasite prevalence should therefore be expected due to low MHC polymorphism.

Larval development of trematodes is reported to be dependent on water temperature [61–65]. An increase in temperature leads to a reduction in the cercarial developmental time and also triggers cercarial release. In a study that tested the effect of temperature changes on the cercarial-shedding rate of two trematodes, the optimum temperature for cercarial emergence was ca 25 °C [65]. In accordance with this, water temperatures being higher in the southern part of Sweden than in the northern, the number of trematodes in beavers was expected to be higher in the South. Russian studies indicate that the intensity of *S. subtriquetrus* is highest in beavers at the age of 7–10 months, and then decreases with ageing, but with a seasonal peak in the autumn [37]. Also in Poland, the intensity was higher among young beavers [24]. For *T. rufus*, the intensity (and the prevalence) was higher in the older beavers [24]. These findings might have a bearing on Swedish beavers.

Limited investigations have been conducted in Scandinavia on the parasites of beavers in recent times. The present study is the first quantitative survey of parasites on beavers living in Sweden and other parts of Scandinavia. Previous studies in Norway and Sweden [40, 41, 48] have only reported occasional findings, and the beavers in a broader study, originating in Norway, were located at London Zoo and in Latvia [7]. This study is based on previously unpublished field-data collected in 1997 and 1998. We consider it important to present these results regarding parasite patterns of Swedish beavers, in order to provide a baseline for further studies in Sweden and elsewhere. The aim of this study was to investigate the parasitic fauna of the Eurasian beaver in a North–South gradient in Sweden. Parasite prevalence and intensity were recorded and analysed in relation to the different age groups of the beaver. For endoparasites, parasite values were also analysed in relation to average, yearly air-temperature. The results may give a perspective for studies of beaver parasites in the future, especially in connection to climate change and wildlife in the circumpolar North [66–68].

## Methods

Thirty beavers were collected from hunters during the normal hunting season, from eight localities in Sweden between 18 April and 14 May 1997, and 5 April and 14 May 1998. The beaver-hunting season in Sweden in the 1990’s started in early October and ended in mid-May, in the regions where it was permitted. In the years concerned, annual beaver harvest in Sweden was estimated at 5000 from a population of 130,000 individuals [69]. The beavers were shot by rifle, in most cases in the head.

**Fig. 3:**
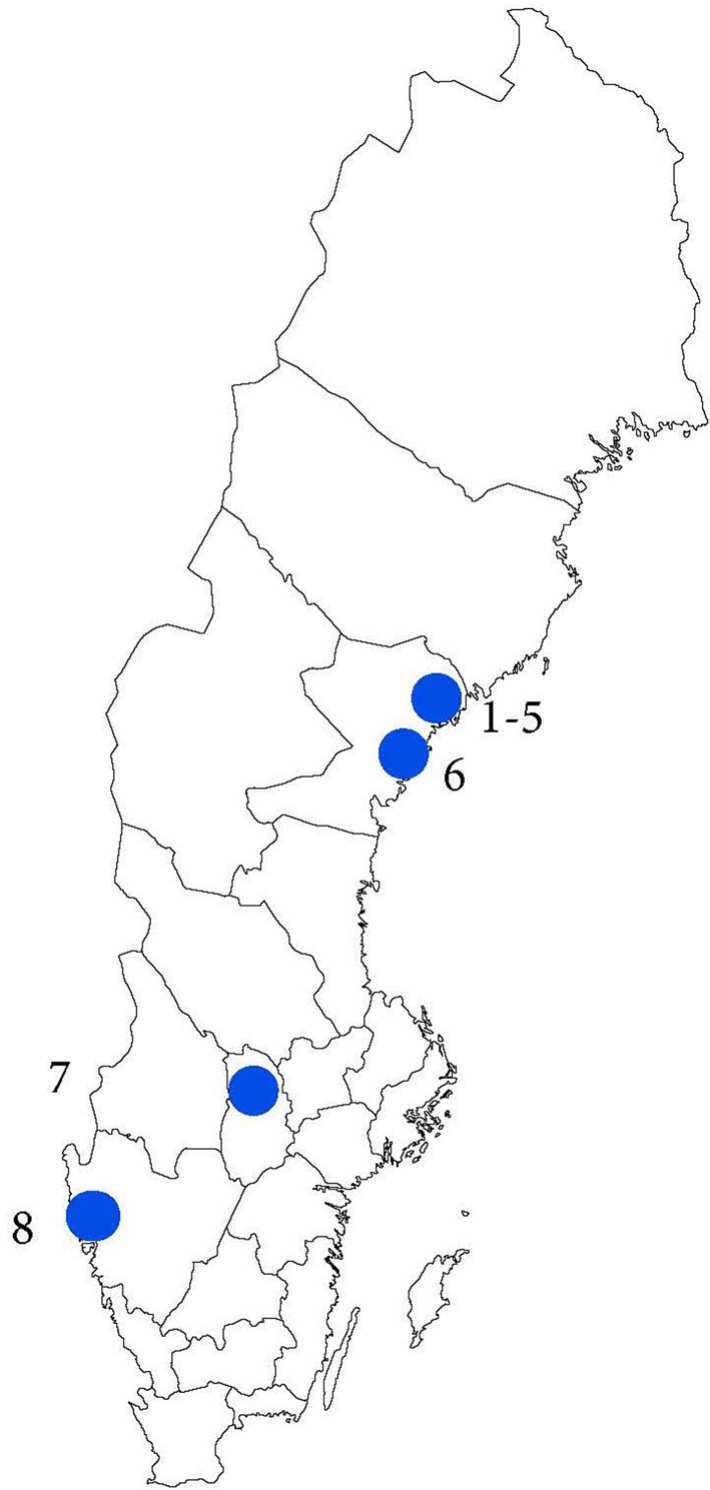
Localities of beavers examined during the hunting season of 1997 and 1998 in Sweden. 1–5: Husån, Gideälven, Moälven, Nätraån and Markumsån; 6: Nordvik; 7: Grimsö; 8: Uddevalla. For details on localities and sample size of beavers, see Table 1

The hunting localities are situated in a North-East to South-West gradient of ca. 700 kms., consisting of a wide range of climate zones (Table 1, Fig. 3). No air or water temperatures were recorded at the respective localities at the time of hunting. To show the temperature air-gradient at the time, records from non-maritime weather stations nearest the hunting locality were gathered from the Swedish Meteorological and Hydrological Institute SMHI. Annual mean temperatures for 1997 (with standard deviation) were calculated from monthly averages estimated by SMHI (values for 1998 were not available for all stations) [70]. Due to lack of weather stations, and minimal distances between hunting localities (Fig. 3) in the North, some of the localities were assigned to the same stations. The annual mean temperatures ranged from 2.9 to 6.7 °C (Table 1). The difference in length of summer between northern and southern localities is 3–4 weeks [70]. Difference in air temperatures and length of summer should be reflected in water temperatures, and air temperature is therefore used as a proxy for water temperature and length of the vegetation period [71].

**Table 1:** Localities with mean temperature, and number of beavers examined for endo- and ectoparasites during the hunting seasons of 1997 and 1998

No	Locality	Latitude longitude	Rivers/lakes	Mean (SD) air temperature 1997, and weather station	Beaver 1997	Beaver 1998	Total
1	Husån	63˚ 34ʹ N 19˚ 03ʹ E	Medium sized river	2.9 °C (8.9) Hemling A 138390	8	1	9
2	Gideälven	63˚ 26ʹ N 19˚ 00ʹ E	Large river	2.9 °C (8.9) Hemling A 138390		1	1
3	Moälven	63˚ 24ʹ N 18˚ 34ʹ E	Large river	2.9 °C (8.9) Hemling A 138390	1		1
4	Nätraån	63˚ 12ʹ N 18˚ 27ʹ E	Medium sized river	3.7 °C (8.2) Västmarkum A 138070	4	1	5
5	Markumsån	63˚ 03ʹ N 18˚ 11ʹ E	Small river	3.7 °C (8.2) Västmarkum A 138070	1		1
6	Nordvik	62˚ 56ʹ N 18˚ 05ʹ E	Two lakes	4.6 °C (8.2) Ullånger 128590		3	3
7	Grimsö	59˚ 44ʹ N 15˚ 28ʹ E	Two small rivers and one lake	5.6 °C (8.0) Kloten 95530		5	5
8	Uddevalla	58˚ 38ʹ N 12˚ 05ʹ E	Two lakes	6.7 °C (7.5) Kroppefjäll-Granan A 82360		5	5
					14	16	30

Most beavers (n = 21) were obtained from rivers and streams, some (n = 9) from lakes (Table 1). Entrails were collected on the day of the kill; however, as sampling took place during the course of hunting, the collection of material was sometimes conducted after intestines were removed or pelts had been tampered with, such that quantitative sampling could at times not be completed for all variables. For one specimen, data on new infections are consequently lacking and, for some beavers, only the presence of adult nematodes (n = 7) or trematodes (n = 5) could be proven, and no counts made. In some cases, data for mites (n = 15) and beetles (n = 13) are also lacking.

### Collection of fur-living ectoparasites

Beaver carcasses were stored overnight, either hung in separate cooling rooms with no contact with walls or floor or contained in plastic bags. The ectoparasites were collected from the floor of the cooling room or from the plastic bag; in addition, and with greater success, a warm hand was placed on the fur, such that the parasites attracted by the warmth migrated to the person’s hand, where they were easily caught. The sampling was terminated when invertebrates ceased to migrate. They were preserved in 70% ethanol. In this way, invertebrates in the fur from 17 beavers from seven localities were examined and determined to species by macroscopic examination. For collection of mites, both ears of 15 beavers from seven localities were sampled and preserved in 70% ethanol, and the parasites found were then counted and determined as to taxa under a 40× stereomicroscope.

### Collection of endoparasites

Lungs and livers of 25 beavers were dissected and examined for helminths using a stereomicroscope. The stomach, small intestine, cecum and colon of all 30 beavers were separated and cut longitudinally to examine for trematodes in the intestinal content and for nematodes in the mucosa. The contents of the stomach, small intestine, cecum and colon were placed in separate buckets, for each organ in the gastrointestinal tract, and 4 L of water added to each bucket. The sample was homogenised and a sub-sample of 0.2 L was taken, except for the cecum from which 0.4 L was taken. The sub-samples were placed in a sieve system (smallest mesh 150 μm) and washed with water. The remains were then examined under a stereomicroscope, and the immature trematodes counted. The number of immature (metacercarian and juvenile) trematodes found in the gastrointestinal tract of 29 beavers, by combining subsamples for each organ, was used as an index. The entire contents of the cecum were sieved and all adult trematodes found were counted in 25 beavers. The infection intensity of adult (in 23 beavers) and immature (in 29 beavers) nematodes respectively, was indexed by counting individuals for 5 min, since the abundance of nematodes in some cases was judged too high for a total count. In a few cases, due to external circumstances, adult nematodes (7 beavers) and trematodes (5 beavers) were not counted, but their presence was recorded. All procedures described above except the nematode index were carried out according to laboratory practice at the National Veterinary Institute, Uppsala, Sweden.

### Age determination

Beavers were aged by investigation of root closure of the teeth [72]. The criteria for the method are degree of eruption, closure of basal openings, and annual cementum layers visible at the base of the mandible molars when sectioned longitudinally. Beavers were divided into five age classes: one (n = 8), 2 (n = 5), 3 (n = 6), 4 (n = 5), and 5 years or older (n = 2). Four individuals were not determined as to age.

Wilcoxon rank-sum tests were performed with JMP software. The significance level was set at P = 0.05.

## Results

Five parasite taxa were identified. Four of these were present in each of the beavers examined; *S. subtriquetrus*, *T. rufus*, *P. castoris* and *Schizocarpus* spp. The fifth species found was a bird flea of the genus *Ceratophyllus*; in a single beaver only one such insect was found. Two non-parasitic organisms were also identified on the beavers. One chironomid larva was found on one beaver and, on another, more than a hundred juvenile annelids of the family *Enchytraeidae*. No helminths were found in lungs or liver.

Beaver beetles, *P. castoris,* were found in the fur of all investigated beavers. The number of beetles varied from 1 to 14 per beaver, except for one with 742 (mean = 48.3; SD = 178.8; n = 17). Two new faunistic provinces for *P. castoris* in Sweden were found, i.e., Dalsland and Västmanland (Fig. 3, localities nos. 8 and 7 respectively). All ears examined were infested with beaver mites, *Schizocarpus* spp., not described to species level. The mean number of mites per beaver (i.e., both ears) was 270.5 (SD = 169.3; n = 15).

**Fig. 4:**
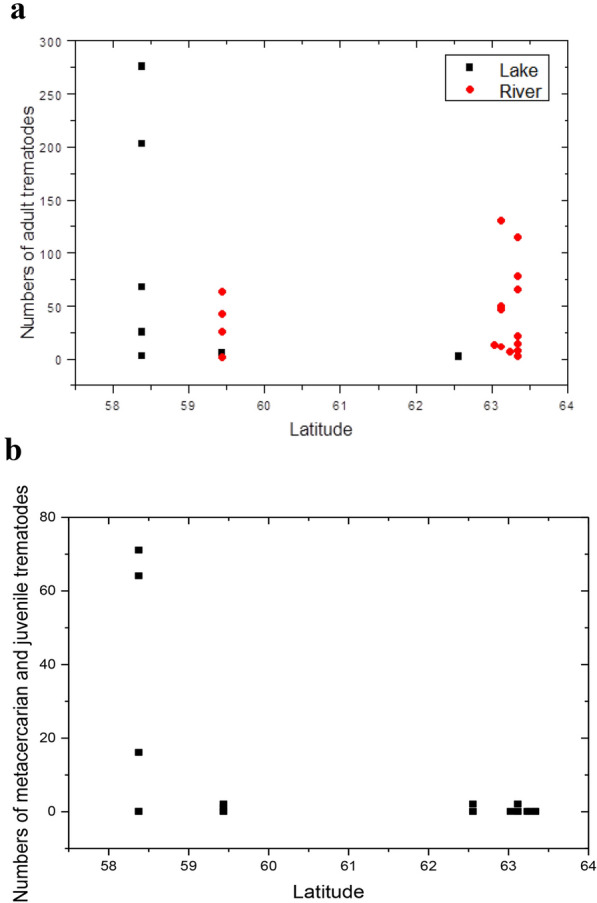
The intensity of trematodes in the studied beavers, over a latitudinal gradient. **a** The intensity of adult trematodes in individual beavers shown in a South to North gradient. Each dot represents one beaver’s intensity of trematodes. Different symbols are used for beavers collected in lakes and rivers; **b** The intensity of new infection by metacercarians and juvenile trematodes of *S. subtriquetrus* shown in a South to North gradient

Mature *S. subtriquetrus* parasitized the cecum of all 30 beavers examined (mean 51.1; SD = 67.6 for the 25 beavers where *S. subtriquetrus* were counted). Six out of these 25 beavers harboured 68% of the total trematode load. Beavers in southern Sweden (latitude 58˚ 38ʹ–59˚ 44ʹ N) tended to have more adult trematodes (mean 71.2, SD = 93,3, n = 10) than those from northern Sweden (mean 37.7; SD = 41.8; n = 15; latitude 62˚ 56ʹ–63˚ 34ʹ N), though the difference was not significant (Wilcoxon rank-sum test, Z = 0.50, P = 0.62, n = 25; Fig. 4a). Immature individuals (metacercariae and juveniles) were found in the small intestine, cecum or colon of 24.1% (7 of 29) of the beavers. The prevalence of metacercariae and juveniles was 50.0% (5 of 10), in beavers from the southern localities (locality 7–8) and 10.5% (2 of 19) in the northern localities (locality 1–6). The infection of immature trematodes was significantly higher in localities 7–8 (mean 15.4; SD = 27,9, n = 10) than in localities 1–6 (mean 0.21; SD = 0,63; n = 19); Wilcoxon rank-sum test, Z = 2.39, P = 0.017, n = 29 (Fig. 4b).

The beaver nematode *T. rufus* parasitized the gastric mucosa of all 30 beavers examined. The intensity of the nematode infection was similar to that of the trematodes. The mean number of adult nematodes was 72.1 (SD = 78.5; n = 23) and the densities of adult nematodes in localities 7–8 (mean 120.4; SD = 95.7; n = 10) was higher than in localities 1–6 (mean 34.9; SD = 31.4; n = 13; Wilcoxon rank-sum test, Z = 2.64, P = 0.0084, n = 23). For juvenile nematodes, no such trend was observed (South: mean 11.6; SD = 11.7, n = 10; North: mean 17.1; SD = 40.6; n = 19; Wilcoxon rank-sum test, Z = 0.115, P = 0.91, n = 29).

Beavers at the age of approximately 1 year had significantly higher infection of adult *S. subtriquetrus* (mean 99.3; SD = 100.1; n = 7) than those of 2 years or older (mean 32.8; SD = 40.9; n = 17; Wilcoxon rank-sum test, Z = 2.10, P = 0.036, n = 24). The young beavers also had significantly more new infections (mean 19.0; SD 30.5; n = 8) than the older ones (mean 0.12; SD 0.49; n = 17; Wilcoxon rank-sum test, Z = 2.54, P = 0.011). One-year old beavers did not have higher infestation of nematodes (neither adult nor juveniles) or beetles as compared to older beavers. For mites, the sampled number of 1-year olds was only three individuals, which was considered insufficient for testing.

## Discussion

All beavers examined in the present study were infected by four parasite taxa—the trematode *S. subtriquetrus*, the nematode *T. rufus*, the beaver beetle *P. castoris,* and beaver mites *Schizocarpus* spp*.* Only two helminth species were thus found and identified, which may be compared with the total reported 33 helminth species from beaver populations that have survived extinction in Europe and Asia [7, 37]. Out of the helminth species reported, several existed in just some of the local beaver populations, suggesting that these are autochthonous and form distinct aboriginal populations [7]. Of the parasites listed in the latter report [7] only two, *S. subtriquetrus* and *T. rufus*, existed in the Norwegian population, from which the Swedish population originates. Beavers from Norway were also exported in 1927 to Latvian rivers and, subsequently, *S. subtriquetrus* and *T. rufus* are found in Latvia. We conclude that all populations originating from Norway have the same restricted helminth fauna. In Poland, where Norwegian beavers were not introduced [73], two studies found four species of helminths, including *T. rufus* and *S. subtriquetrus* [23, 24]. In these studies, 43 individuals [23] and 48 individuals [24] were sampled, respectively. In Lithuania, likewise, in two beavers of non-Norwegian origin [73], only *T. rufus* and *S. subtriquetrus* were found [22].

The most remarkable finding in our study was the exceptionally high prevalence (100%) of each of four parasites found. To our knowledge, no other study of beaver parasites has shown such a high prevalence, although high numbers, in particular for *S. subtriquetrus,* have been found elsewhere. In Poland a 93% prevalence was found with *S. subtriquetrus* and 63% with *T. rufus* (n = 43) in one study [23], and similar figures, 93.7% and 68.7%, respectively, (n = 48) were found in another study [24]. Belarusian beavers displayed a 90.7% prevalence for *S. subtriquetrus*, and 69.3% for *T. rufus* [27]. In a study of beavers in the Czech Republic, the prevalence for *S. subtriquetrus* was 82% (9 out of 11 animals) [35]. Relatively high prevalence of helminths has also been found in *C. canadensis*, as high as 83% and 84%, respectively, for *T. americanus* and *S. subtriquetrus* [15]. In other studies, a prevalence of 89% [12] and 83% [14], respectively, was found for *S. subtriquetrus*.

*Platypsyllus castoris* had a prevalence of 100% in the 17 investigated beavers. This may be compared to the 60% prevalence from 45 living and dead beavers reported in a North American study [42]. The prevalence of *Schizocarpus* spp., sampled from the ears of 15 beavers, was also 100%. Prevalence of the latter taxa are not usually reported in the literature and may normally be high.

The 100% prevalence of the parasite species found (*T. rufus*, *S. triquetrus*, *P. castoris* and *Schizocarpus* spp*.*) might be explained by the two events experienced by the Swedish beaver population—the population bottleneck in the original Norwegian population, and the sampling effect in the reintroduction process. A genetic study of Swedish beavers in the early 1990s showed low or no genetic variation [4]. The parasite species that passed through the aforementioned events might have gained from the low genetic diversity within the host population. Heterozygosity in MHC genes allows presentation of a wider range of pathogen-derived peptides, and thus provides greater resistance to infection [74, 75]. Individuals with MHC variants preventing infection may have a large competitive advantage over parasitised conspecifics, even if the parasites do not have a large effect on survival in uncompetitive situations. This would cause strong selection on MHC genes without much effect on population dynamics, which in turn may be determined mainly by the carrying capacity of the environment [74]. In the investigation of the MHC class II gene *DRB* of the Eurasian beaver, it was found that all populations, except one, were monomorphic [60]. The data in that study [60] corresponded with the results of the restriction fragment length polymorphism (RFLP)-based survey that found monomorphism of the *DRB* gene and other MHC loci in our Scandinavian population [4]. It has been argued that immune response to parasites may be explained by the variation in MHC genes [76]. In the same study [76], it was proposed that parasites coevolving with their hosts have had a major influence on MHC polymorphism. In a study of the genetic diversity of micro-satellites in *C. fiber,* the population in Finland, which like the Swedish derives from Norway, had low heterozygosity as well as allelic richness, compared with other populations [5]. It was suggested that this may limit the breadth of the immune function [5]. This also implies that Swedish beavers may have a reduced resistance to infections of parasites and diseases, which could explain the high prevalence of parasites in the population.

Beavers in the southern (localities 7 and 8) tended to have more adult *S. subtriquetrus* than those in northern localities 1–6. We also found significantly more new infections of the trematode in beavers in the southern (localities 7–8) than in the northern (localities 1–6), indicating that these are exposed to more recurrent infections. We interpret the difference in recurrent infection to depend on the climate differences influencing water temperature between northern and southern Sweden (Table 1). Annual means for air temperatures, based on monthly averages for weather stations close to the localities, clearly show the climate difference between localities 1–6, and 7–8, respectively. Although there is a North–South gradient, the two groups are clearly separated. We assume that this climate difference is reflected in water temperatures. Our result is in accordance with other studies [61–65], demonstrating the influence of water temperature on release of cercariae from the intermediate hosts. Parasitic eye flukes in fish (*Diplostomum* sp*.*) are more abundant in water bodies receiving heated cooling-water from a nuclear power station, as these have a higher temperature than the surrounding lakes [62]. In Sweden there is a North–South species diversity gradient of freshwater gastropods [71, 77] responding to the climate zones. *Stichorchis subtriquetrus* has a large number of intermediate hosts [18]. The larger number of intermediate host species in South Sweden could contribute to the higher number of new infections. A possible confounding factor could be that trematode dispersal may be more effective in lakes, with calm water, and as the northern localities have rivers, and the southern mostly lakes, this could produce the geographic difference. There are also more potential intermediate host species in lakes [71]. On the other hand, the number of new infections were low in both lakes and streams in the North (Fig. 4b). In addition, adult, but not juvenile, *T. rufus* had a higher infection rate in the South of Sweden. In terrestrial systems, the development of nematode eggs and larvae are shown to increase with temperature, allowing higher transmission speed [67, 68, 78, 79]. This phenomenon may also occur in the warmer climate in southern Sweden and be relevant for the transmission of parasites among beavers.

The youngest beavers, 1-year-olds, were more severely infected with both adult and juvenile *S. subtriquetrus* than the older ones. This is in accordance with Russian studies, using the release of eggs from live beavers over seasons as an index, where abundance index increased gradually from new-born kits, as infections built up to the highest values of mature *S. subtriquetrus* in young beavers (7–10 months) [37]. With increasing age, the trematode abundance index then decreased. There was however also a seasonal cycle with low intensity in spring and a peak in autumn [37]. Our sampling was made during a period of 6 weeks in springtime, allowing us to make a snapshot comparison of age classes, where the youngest beavers all were close to 1 year old, since they were born in the spring of the previous year. Studies from Poland appear to be contradictory, where one study (n = 48) showed that beavers up to 2 years of age had twice as high infection by numbers of *S. subtriquetrus* as beavers older than 2 years [24], while an earlier study (n = 43) in a nearby area did not show any difference between the host’s age groups [23]. In that study, however, the youngest age group consisted of individuals 1 year and younger. Since the material probably was collected over several seasons, some of these individuals may have been kits which may not yet have been fully infected [23]. In a study of *C. canadensis* helminth fauna in central Texas (n = 36), yearlings (1–2 years of age) had a significantly higher burden of *S. subtriquetrus* than adults (> 2 years old) and harboured more than 90% of the total *S. subtriquetrus* counted, while kits (< 1 year) had no infections at all [14]. The general pattern, thus, appears to be that *S. subtriquetrus* infections are built up during the beavers’ first year of life and then receded (with possible seasonal fluctuation), although this has not been shown in all studies [15, 35].

In the present study, no difference was found for adult *T. rufus* between young and older beavers. In two Polish studies however, both a higher prevalence and a higher infectivity was seen in adult beavers, although, as mentioned above, age classification differed [23, 24]. The authors did not explain the age difference, but apparently, they demonstrated a building up of parasites over years even though numbers decreased in autumn, probably due to expulsion of adults and inhibiting of larval development [24]. They also speculate that *T. rufus* numbers may vary with beaver population density, since infections had increased between the two studies [24]. There is thus a possibility that beaver populations in Sweden have a lower density of beaver individuals and that this affects host-parasite dynamics.

## Conclusions

Beavers in Sweden harboured a small number of parasite species, which mirrors the impoverished parasite fauna of the original Norwegian population. At the same time, the prevalence of both trematodes, nematodes, arthropods and coleoptera were high, which may be due to low MHC polymorphism caused by the population bottlenecks. Beavers from the southern localities in Sweden, where higher temperatures prevail, had more new infections of trematodes and higher adult nematode intensity than those from the northern localities. The youngest beavers had a higher infection of trematodes as compared to older beavers, but no such effect was noted for nematodes or coleopterans.

## Data Availability

The datasets used and/or analysed during the current study are available from the corresponding author on reasonable request.
